# Chitosan-Based Edible Coatings Containing Essential Oils to Preserve the Shelf Life and Postharvest Quality Parameters of Organic Strawberries and Apples during Cold Storage

**DOI:** 10.3390/foods11213317

**Published:** 2022-10-23

**Authors:** Paul-Alexandru Popescu, Laurentiu Mihai Palade, Ioana-Cătălina Nicolae, Elisabeta Elena Popa, Amalia Carmen Miteluț, Mihaela Cristina Drăghici, Florentina Matei, Mona Elena Popa

**Affiliations:** 1 Faculty of Biotechnology, University of Agronomic Sciences and Veterinary Medicine of Bucharest, 011464 Bucharest, Romania; 2 National Research & Development Institute for Food Bioresources-IBA Bucharest, 6 Dinu Vintilă Street, District 2, 021102 Bucharest, Romania; 3 Research Center for Studies of Food Quality and Agricultural Products, University of Agronomic Sciences and Veterinary Medicine of Bucharest, 011464 Bucharest, Romania

**Keywords:** edible coatings, chitosan, shelf life, strawberries, apples

## Abstract

Edible coatings and films have been researched for more than three decades due to their ability to be incorporated with different functional ingredients or compounds as an option to maintain the postharvest quality of fruits and vegetables. The aim of this study was to evaluate the effect of three types of chitosan-based (CH) edible coatings obtained from medium and high molecular weight chitosan, containing ascorbic or acetic acid and sea buckthorn or grape seed essential oils on the physical–chemical and microbiological properties of organic strawberries and apple slices during cold storage at 4 °C and 8 °C. Scanning electron microscope images showed both a smooth structure and a fracture and pore structure on strawberry coatings and a dense and smooth structure on the apple slices coatings. Further, the edible coatings managed to reduce the microbial load of yeasts and molds of the coated strawberries during the storage period. Overall, the treatments preserved the ascorbic acid, total polyphenol content, and antioxidant activity for all the tested samples compared to the control sample, throughout the storage period. In addition, the water activity (a_w_) of the coated samples presented lower values (0.96–0.98) than the control samples. The obtained results indicate that the developed chitosan-based edible coatings could maintain the postharvest parameters of the tested samples, also leading to their shelf-life prolongation.

## 1. Introduction

Food products are vital for the survival of human beings and, over time, consumers have become more demanding in terms of their quality. However, food products are naturally perishable and can be degraded by environmental factors, such as fungal or bacterial contamination [1,2]. Therefore, it is essential to protect them from spoilage microorganisms in order to increase their shelf life and satisfy consumer demands [3].

Conventional food packaging materials are made from petroleum-based polymers and have been used to protect food products and maintain their quality and sensorial attributes [4,5]. Due to their flexibility, lightness, and transparency, these films have been massively used, rendering the achievement of modified atmosphere packaging (MAP) for commodities that are highly perishable [6,7]. Innovative packaging methods, such as edible coatings, polymeric films, or modified atmosphere packaging, are used more and more in order to maintain the quality of fresh-cut produce during the transport and distribution phases [8]. When gas is flushed into the pack or the produce receives no alteration of the atmosphere, along with the permeability properties of the employed packaging, this allows equilibrium MAP (EMAP) to be reached [6,9]. However, this has led to a serious ecological problem because of their nonbiodegradability [10,11]. Their complete replacement by biodegradable and sustainable packaging materials is currently being studied and is desired by both the industry as well as the final consumers [12]. Edible coatings and films are among the newly developed biodegradable packaging materials. EU regulations EC 1935/2004 and EC 10/2011 comprise specific recommendations with regard to the declaration of conformity that must accompany food commodities; these provide guidelines on the use of packaging materials (active and intelligent), release of substances considered food additives thereof (must be accredited), as well as materials used during processing (i.e., direct or indirect contact with food along the production chain) [13,14]. The main advantage of edible films and coatings compared to traditional synthetic packaging is that they are safe for the environment and the consumer [15,16,17]. They consist of a thin layer of a nontoxic, edible surface applied to the food product, with or without subsequent removal, with the objective of extending the shelf life and quality of the product [7,18]. 

Edible coatings and films are produced from polymers derived exclusively from renewable resources and applied as a liquid form directly on the surface of food [19,20]. At the compositional level, a material is used as the structural matrix of the edible coating and bioactive compounds, nano-composites or natural extracts are added to improve the functional properties of the coating [21]. Arguably, edible coatings pertaining a certain thickness along with selective permeability may alter the inner atmosphere composition of the package without gas filling/flushing, rendering them EMAP coatings and may avoid further packaging [6,22]. Most recently, Jalali et al., 2020 developed an integrated mathematical modelling as a guide for strawberry shelf-life prediction under realistic conditions by taking into consideration the transpiration and respiration rate. The model simulated the strawberries shelf life under open tray conditions and MAP conditions, taking into consideration the temperature and humidity as test parameters and allowing the optimization of the supply chain [23]. Each material is chosen according to the characteristics of the food to be coated [24,25]. For example, studies show that fried food products can be coated with cellulose-based edible films because they prevent oil absorption [26], meat products can be treated with alginate edible films because of the delays of the lipid oxidation process [27] or chitosan base edible films in order to preserve the color and prevent lipid oxidation [28]. Pectin-based edible films can be used on fruits and vegetables in order to maintain the color, prevent weight loss, and reduce microbial growth [29]. Edible films and coatings are defined as thin layers applied to foods to protect them, to extend their shelf life and to improve certain qualitative properties such as the appearance. They are created from edible and natural renewable sources (proteins, polysaccharides, lipids) and contain anti-oxidants, anti-browning agents and colorants. Sometimes additives are added to improve the efficiency of the coating; by themselves or in combination with additives, they can help prevent drying out, immobilize microbes, or reduce access to oxygen [30,31,32]. Different methods of application of coatings are used: spraying, brushing or electro-spraying [4]. Several polysaccharides, such as starch, alginate, chitosan, pullulan, pectin and carrageenan are currently being used by the researchers to produce edible coatings [33]. 

Chitosan is a well-known biopolymer used for several years in different sectors of the industry and is known for its antibacterial [34,35] and antifungal properties [36,37,38]. The main advantages when using chitosan as matrix for a packaging material, beside its antimicrobial capabilities, is that it is biodegradable, thus being a sustainable choice for the food industry [39,40]. Chitosan, a deacetylated derivative of chitin, is nowadays used as a biodegradable food packaging material because of its great film-forming and good antimicrobial properties [41]. Other properties that make chitosan a good packaging material are its ability to be cross-linked in order to avoid dissolution in acidic solutions, high porosity, good hydrophilicity and big adhesion area [24]. In addition to these properties, chitosan is mainly produced from see-food industrial waste, thus being a sustainable material and that has excellent functional properties when combined with other materials. Chitosan can be used as packaging film or as coating applied directly on the food product [41].

For several years now, there has been a growing demand for fresh fruits and vegetables, especially for minimally processed ones due to consumer needs for fresh, healthy snacks for their busy lives. However, these foods are more vulnerable to damage and loss of market quality [31,32]. For this reason, many methods are used to address these advantages and allow freshly-cut products to have a longer shelf life than the 2–9 days assuming proper temperatures are assured (cold storage) [42]. The use of edible coatings is one of them. These consist of using a material for coating and adding natural antimicrobials to preserve the quality and improve the shelf life of the food product. This method has already been studied on different fruits and vegetables [3]. 

With more than thirty years of scientific insight gathered, currently, various studies are conducted worldwide to address the need to improve the properties of edible coatings and films to develop an alternative to synthetic polymers and hazardous chemicals [19,24,25]. Moreover, by knowing the composition of the food coating, the organoleptic properties and the shelf life of the food can be improved. Edible coatings/films have become the subject of many research topics in the food industry, especially on fruits, such as strawberries [43,44,45], apples [46,47], cherries [48,49], kiwis [50], melons [51], and pears [52]. 

Strawberries are fruits that possess a very short shelf life because of their high metabolic activity and water content and the susceptibility of being contaminated with fungal strains, especially grey mold (*Botrytis cinerea*). Physical injuries can also occur in different stages of the traceability chain because of their soft texture and lack of protective rind [53]. The main postharvest technique used in the industry to extend the shelf life of strawberries is refrigeration at temperatures of 0 °C [54]. With superior barrier attributes, edible coatings are intended to prolong the shelf life and exhibit a significant effect as postharvest treatment on fresh-cut fruit; by maintaining the physiological and physicochemical properties of the produce, they are considered to attain efficiency and functionality towards the retention of postharvest quality [7]. In recent years, edible coatings have been used to extend the shelf life of strawberries by delaying the textural changes, reducing the respiration rate, and providing a gas permeability barrier, thus maintaining the sensorial and nutritional values at higher levels [55]. Edible coatings made from soy or wheat gluten with incorporated thymol and calcium chloride were applied on fresh strawberries in order to analyze the improvements of physical–chemical and microbiological properties of the coated samples. The tests were carried out over a 9-day period, and the appearance of the samples remained unchanged. The firmness, ascorbic acid content, total soluble solids, and total sugars were maintained over the storage period in comparison to the control sample. The coated samples with thymol and soy protein or white gluten recorded higher chroma hue angle values and the lowest values of anthocyanin content [53].

Four types of edible coatings made from low methoxyl pectin (LMP), carboxymethyl cellulose (CMC), Persian gum (PG), and tragacanth gum (TG) were developed and applied on fresh strawberries in order to test their shelf life improvement capacity. A physical–chemical analysis, such as weight loss, ascorbic acid, total phenolics, and anthocyanins content were evaluated over a period of 16 days of storage at 4 °C. The results show that the coated samples presented better sensory attributes compared to the control samples. The strawberry samples coated with the CMC solution had reduced decay and weight loss, as well as better values of the physicochemical analysis [56]. 

The antifungal properties of chitosan/glycerol films were tested over *E. coli*, *S. aureus,* and *B. cereus* using the disc-diffusion technique by Salvia-Trujillo et al. (2015) [47]. When coated with a chitosan/glycerol 30% solution [55], the strawberry samples had an improved shelf life, with excellent antibacterial and antifungal activity (1 week). Moreover, it maintained the sensorial properties of the strawberries, with no alteration in appearance, texture, and flavor [55]. 

Fresh-cut apples are also vulnerable to microbial and enzymatic spoilage because their natural protective barrier is removed, and the internal tissue, which is an excellent source of nutrients, is exposed to external factors [57]. Apples are usually resistant to microbial decay because of the presence of phenolic and flavonoid compounds, such as catechins, phloridzins, tannins, and chlorogenic acid, on the apple peel [58]. There are several studies that researched the effect of different chitosan edible films on apples or fresh-cut apples, the results highlighting that the edible films managed to reduce the respiration rate [59,60], the microbial load [57,59,61], and the weight loss [57,60,62]. 

The scope of this research paper was to evaluate the impact on the shelf life and nutritional values of three newly developed chitosan-based edible coatings on strawberries and apple slices. The chitosan-based edible coatings were obtained as follows: 2% medium molecular weight chitosan and 1% acetic acid; 2% medium molecular weight chitosan and 1% acetic acid; and 1% high molecular weight chitosan and 2% ascorbic acid. These formulations were enriched with grape seed or sea buckthorn essential oils; these were chosen over a previous study performed by the same authors in which the EOs were analyzed for their antifungal properties [63]. 

## 2. Materials and Methods

### 2.1. Biological Material—Strawberries and Apples

Organic strawberries (*Fragaria* × *ananassa*) and apples (*Malus domestica cv Florina*) were purchased from a local market in Bucharest (Romania) and transferred to the laboratory within 2 h after purchase. The biological material was kept overnight at refrigerated temperatures (4 °C) before the coatings were applied. The next day, the fruits were visually inspected for decay or damage. Fruits free of physical injuries were selected for the further treatment taking into account the similarity in shape, color, and size.

### 2.2. Materials

Medium molecular weight chitosan with a deacetylation degree of 75–85% (448877) and high molecular weight chitosan with a >75% deacetylation degree (419419) were purchased from Sigma-Aldrich (Burlington, MA, USA)and used as received. Ascorbic acid (AC05150) and acetic acid (AC0344) were purchased from Scharlau (Spain), and Tween^®^ 20 (Polysorbate) (97062-332) was purchased from VWR (Radnor, PA, USA) and used as received. Grape seed essential oil was purchased from Herbavit (Bucharest, Romania), and sea buckthorn essential oil was purchased from Hofigal (Bucharest, Romania) and used as received. Folin–Ciocalteu reagent (F9252), xylene (214736), sodium bicarbonate (S6014), sodium acetate (S8750), 2,2-diphenyl-1-picrylhydrazyl (DPPH) (247642), and potassium chloride (P3911) were purchased from Merck (Darmstadt, Germany).

### 2.3. Preparation and Characterization of Chitosan Coatings

Edible coating chitosan-based solutions were prepared by dissolving medium molecular weight chitosan 2% *w*/*v*, high molecular weight chitosan 1% *w*/*v* in acetic acid or ascorbic acid solutions, under constant stirring on a stirring plate (Fisherbrand, Isotemp, China) for approximately 1 h at a constant temperature of 80 °C. Subsequently, the obtained solutions were left to cool at room temperature for 1 h. In order to ensure the mixing of the essential oils with the chitosan solutions, 2 mL of Tween 20 was used as emulsifying agent and then stirred for 5 min. Grape seed (7.5%) or sea buckthorn (7.5%) essential oils were added to each coating solution and then stirred again for 15 min in order to completely emulsify. Composition and coding of the obtained edible coating materials are described in Table 1. The surface morphology of the coatings was investigated using a scanning electron microscope (SEM), FEI Inspect S50 Electron Microscope (FEI, Hillsborough, OG, USA).

### 2.4. Coating Treatment

Before applying the coating treatment, the chosen strawberries and apples were rinsed for 1 min with distilled water and then stored for 1 h at room temperature in order to ensure full water evaporation. The strawberries were coated as such (whole fruit), whereas the apples were cut into slices of the same dimensions. The control samples consisted of fruits without any coating applied. 

**Figure 1:**
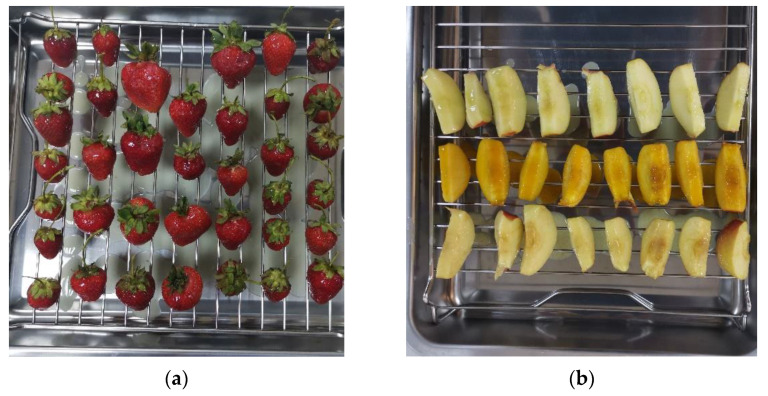
(**a**) Visual aspect of the coated strawberries during the drying process; (**b**) Visual aspect of the coated apple slices during the drying process.

The edible coatings were used to treat the strawberries and apple slices through dipping method. The fruits were dipped into the coating solutions for 20 s and then dried at room temperature for 1 h to remove excess solution from fruit surface (Figure 1). After the coatings were fully dried, the strawberries and apple slices were packaged in perforated polyethylene terephthalate (PET) containers (each container had approximately 80 g of fruit sample) and stored at two distinct temperatures (4 °C and 8 °C). The physicochemical and microbiological properties were evaluated for a period of 7 days.

### 2.5. Scanning Electron Microscopy (SEM) Assay

Apple and strawberry fruit samples coated with chitosan layer were cut into small pieces, approximately 5 × 5 mm in dimension, and fixed on the support stub using carbon adhesive tape. Samples were scanned using FEI Inspect S50 Electron Microscope, in low vacuum mode. Images were acquired at a pressure of 220 Pa and magnification of 200× and 400×.

### 2.6. Dry Matter

The dry matter and humidity were determined by using a thermobalance (RADAWAG MAC 50, Poland). Briefly, five (5) grams of sample were distributed homogeneously in a thin layer on the weighing plate, to obtain reproducible results. Following treatment at 105 °C, the amount of dry matter was determined. All measurements were performed in triplicate.

### 2.7. Water Activity (a_w_)

Strawberries and apple slices (approximatively 80 g per treatment) were homogenized using a stomacher (Interscience, BagMixer 400, Saint Nom, France). The water activity content of the samples was determined using a water activity meter (NOVASINA, LabMaster-aw, Lachen, Switzerland) with an accuracy of ±0.0030 aw within the adjustment range (https://www.novasina.ch/produkt/labmaster-aw-neo/, accessed on 15 October 2022). The samples were placed in containers and allowed to reach the device’s temperature in the thermostatic chamber before reading.

### 2.8. Determination of Total Polyphenol Content (TPC)

An ethanolic (75%) extraction of polyphenols in a sample:solvent ratio of 1:5 was employed by macerating samples for 48 h in the dark at room temperature. After filtration, the resulting ethanolic extracts were collected and stored at −20 °C until further analysis. TPC was determined using the Folin–Ciocalteu method [64] adapted to microscale [65]. Briefly, 20 µL ethanolic extracts was mixed with 1580 μL of distilled water, plus 100 μL of Folin–Ciocalteu reagent and vigorously stirred. After 1 min, 300 μL of aqueous sodium carbonate 20% was added, and the mixture was vigorously stirred again and allowed to stand at room temperature in the dark, for 2 h. Absorbance was measured at 765 nm (on a Thermo Helios Alpha UV/Vis Spectrophotometer, Waltham, MA, USA); TPC was calculated from a calibration curve, using gallic acid as standard. The results were expressed as mg gallic acid equivalents (mg GAE)/L.

### 2.9. Evaluation of Antioxidant Activity (AA) using the DPPH Method

Antioxidant activity was determined by evaluating the free radical scavenging effect on the 1,1-dipheny l-2-picrylhydrazyl radical (DPPH) as described by Villaño et al. (2007) with slight modifications [66]. Briefly, an aliquot of 0.5 mL ethanolic extracts was added to 1.95 mL of DPPH solution (60 μM in ethanol), vortexed, and the absorbance was read at t = 0 (A 515(0)) and t = 30 (A 515(30)) min using a Thermo Helios Alpha UV/Vis Spectrophotometer. The AA was calculated from a calibration curve, by plotting %∆A515 against known quercetin concentrations (3–50 μM), where

%∆A515 = [(A 515(0) − A 515(30))/A 515(0)] × 100.

The results were expressed as μM quercetin equivalents (μM QE).

### 2.10. Determination of Ascorbic Acid Content

The ascorbic acid content was evaluated using the indophenol–xylene extraction method [67]. Briefly, 10 g of sample was mixed with 20 mL of 2% (*w*/*v*) oxalic acid, ground in a mortar, and brought to 100 mL (final volume) with 2% (*w*/*v*) oxalic acid. After resting for 10 min at room temperature and filtration, 2 mL of the content was mixed with 1 mL of 2% (*w*/*v*) oxalic acid, 5 mL of sodium acetate solution, 2 mL of indophenol colorant, and 20 mL of xylene, followed by centrifugation for 20 min at 4 °C and 9000 rpm. The absorbance of the resulting sample extracts was measured at 500 nm, and the results were expressed as mg ascorbic acid/100 g sample (fresh weight).

### 2.11. Microbiological Assay—Molds and Yeasts

Sample preparation for mold and yeast counts was achieved in aseptic conditions (laminar flow, gloves, and scalpels), for each replicate, based on the method described by Jafari et al. (2021) [68] and Eshghi et al. (2022) [69]. Briefly, 10 g of sample was mixed with 90 mL of sterilized distilled water in 100 mL Erlenmeyer flask. One (1) mL of each of the appropriate serial dilution was plated by standard microbiological pour plate technique on malt extract agar (MEA) medium (Scharlau, Spain). All plates were incubated for 5 days at 25 °C, and results were expressed as total number of colonies per dilution. All microbiological determinations were performed in duplicate. 

### 2.12. Statistical Analysis

The study assessed strawberry and apple fruit samples selected from 3 treatments. The DM%/a_w_/TPC/AA/Vit C levels registered by each fruit type were measured over time. All data are expressed as mean ± standard error of the mean (SEM). Results were submitted to JMP 11 Statistical Discovery^TM^ from SAS. Repeated measurement analysis of variance (ANOVA) was performed to investigate the statistical differences among groups for all analyzed parameters employing the Standard Least Squares method. The model effects were treatment (Control, MMC-AcA-GSEO, MMC-AcA-SBEO, or HMC-AsA-GSEO), time (day 0, day 3, day 5, day 7, and day 9), and their interaction (Treatment × Time), in order to evaluate the overall treatment effect, measured at 4 °C and 8 °C. Values of *p* < 0.05 were considered significant. 

## 3. Results and Discussion

### 3.1. Scanning Electron Microscopy (SEM) Assay

**Figure 2:**
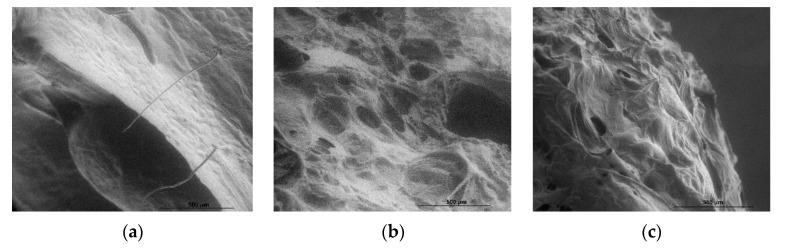

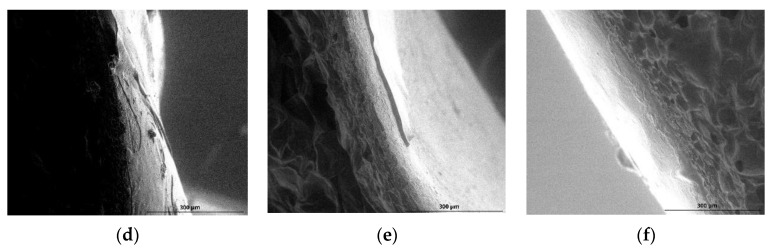
Scanning electron microscopy (SEM) images of (**a**) strawberry coated with medium molecular weight chitosan, acetic acid, and grape seed EO (magnification 500×); (**b**) strawberry coated with medium molecular weight chitosan, acetic acid, and sea buckthorn EO; (**c**) strawberry coated with high molecular weight chitosan, ascorbic acid, and grape seed EO; (**d**) apple slices coated with medium molecular weight chitosan, acetic acid, and grape seed EO (magnification 500×); (**e**) apple slices coated with medium molecular weight chitosan, acetic acid, and sea buckthorn EO; and (**f**) apple slices coated with high molecular weight chitosan, ascorbic acid, and grape seed EO.

Figure 2 shows the morphology of the applied coatings on whole strawberries and apple slices. When analyzing strawberry coatings, fractures and pores were observed in the coatings of fruits dipped in MMC-AcA-SBEO (Figure 2b) and HMC-AsA-GSEO (Figure 2c), while the MMC-AcA-GSEO (Figure 2a) coating showed a continuous and smooth structure. However, the coatings applied on strawberries appeared to be very thin, and consequently, the thickness could not be measured on the coated fruit. The presence of porosity in a sponge-like structure may have occurred due to the structure-elevated roughness associated with the evaporation of the oil; this could be attributed to the disruption of the crosslinking of the chitosan film (C–H bonds) due to the hydrophobic nature of the essential oil components [45]. Similar effects were observed also by Oberlintner et al. (2021) in a biodegradability study of active chitosan biopolymer films enriched with *Quercus* polyphenol extract where a reduced thickness of the chitosan matrix occurred, but no notable difference in morphology was observed [40]. Further, the structure of the chitosan-based coatings appeared dense and smooth on apple slices, the thickness varying from 4.43 ± 0.5 µm on the tissue of the sliced sample to 13.61 ± 0.5 µm on the apple peal. This difference may result from the high humidity of the slice tissue.

### 3.2. Effect of Edible Coating on Fruit Quality Parameters in Strawberries

#### 3.2.1. Dry Matter and Water Activity

**Figure 3:**
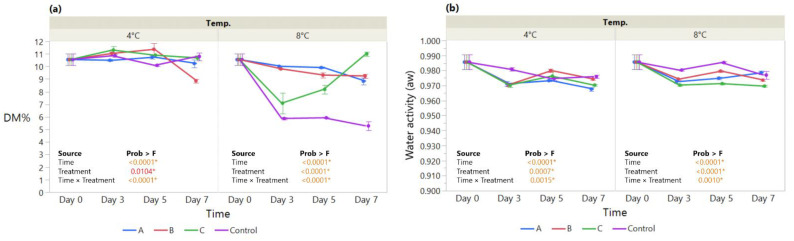
The effect of coating treatment on strawberry (**a**) Dry matter (DM%) and (**b**) Water activity (a_w_) during a 7-day storage period at 4 °C and 8 °C. The *p*-values for the effects of Treatment (coating), Time and Interaction Treatment × Time are shown. Data are means ± SEM (*n* = 3). Asterisk (*) indicates significance (*p* < 0.05*).

The main effects of treatment, time, and their interaction were assayed for the dry matter (DM%) and water activity (a_w_) at 4 °C and 8 °C in strawberries during a storage period of 7 days. DM% measured in coated fruit stored at 4 °C did not vary significantly until day 5 (10.1–11.3%) compared with the control, except for MMC-AcA-SBEO, which showed a significant drop at day 7 (Figure 3a). By contrast, repeated measurement analysis revealed different patterns for coated strawberries stored at 8 °C when compared to 4 °C. The improvement in DM% for both MMC coatings over the control was displayed by the significant effects of Treatment (*p* < 0.0001*) and Time × Treatment (*p* < 0.0001*). The HMC-AsA-GSEO revealed a drop at day 3 (7.09%), followed by a significant increase toward day 7 (11.01%) compared with the control.

In terms of water activity (a_w_) measured at 4 °C and 8 °C, the improvement for coated fruit was displayed by the significant Treatment effect (*p* = 0.0007* at 4 °C and *p* < 0.0001* at 8 °C) (Figure 3b). Over time, the coated fruit maintained overall a_w_ levels below the control, as displayed by the Time × Treatment interaction effect (*p* = 0.0015* at 4 °C and *p* < 0.0010* at 8 °C), except for MMC-AcA-SBEO (treatment B) at 4 °C on days 5 (0.98) and 7 (0.97).

Provided that moisture transfer occurs between the fruit produce and the surrounding environment, bound water become a function of time throughout cost–storage [70]. Water activity values of strawberries (Figure 3b) are maintained between 1.00 and 0.95, a range that favors microbial spoilage of fresh fruit [71,72]. Our results show definitive overall improvement at both 4 °C and 8 °C for coatings over the control and are in line with previous findings [72,73,74]. 

In conjunction with the DM% evolution, the weight loss of strawberries, resulting from the loss of water content, are consistent in terms of the improvement due to coating application. 

Given that water activity regulates moisture migration, differences in DM% were drastic for HMC-AsA-GSEO, whereas the MMC exhibited steady trends throughout storage. This might be attributed to the difference in coating permeability, which in turn accounts for the amount of water available for chemical and physical reactions [72]. For example, Choi et al. (2016) observed that the excessive addition of EO altered the structure of an HPMC (Hydroxy Propyl Methyl Cellulose) coating, resulting in a loosened structure, more prone to water migration [75]. 

#### 3.2.2. Total Polyphenol Content, Antioxidant Activity, and Vitamin C Content

**Figure 4:**
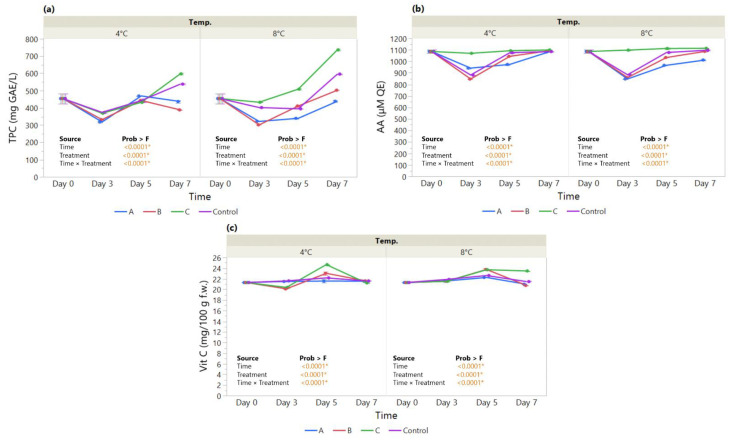
The effect of coating treatment on strawberry antioxidant status during a 7-day storage period at 4 °C and 8 °C. (**a**) Total Polyphenol Content (TPC—mg GAE/L); (**b**) Antioxidant Activity (AA—μM QE); and (**c**) Vitamin C content (Vit C—mg/100 g fresh weight). The *p*-values for the effects of Treatment (coating), Time and Interaction Treatment × Time are shown. Data are means ± SEM (n = 3). Asterisk (*) indicates significance (*p* < 0.05*).

The effect of an edible coating on total polyphenol content, antioxidant activity, and vitamin C content was assayed in strawberries over a 7-day storage period. Our results show a significant drop in TPC measured at 4 °C on day 3 followed by an increase on day 5 irrespective of the coating. At day 7, only the HMC-AsA-GSEO increased progressively during storage at 4 °C (597 mg GAE/L), revealing a significant improvement over the control (Figure 4a). The significant main effect of coating Treatment (*p* < 0.0001*) and Treatment × Time (*p* = <0.0001*) is also displayed at 8 °C by the similar patterns in TPC over time, with HMC-AsA-GSEO registering higher levels than the control throughout storage (454.4–736.8 mg GAE/L). 

Similarly, the antioxidant activity (AA) shows close patterns over time between the MMC coatings and the control measured both at 4 °C (Treatment × Time, *p* < 0.0001*) and 8 °C (Treatment × Time, *p* < 0.0001*) (Figure 4b); the control and the MMC coatings registered a drop at day 3 at both temperatures, while the HMC-AsA-GSEO displayed steady levels maintained until day 7. At the same time, the vitamin C content (Figure 4c) measured at 4 °C (20.11–24.67 mg/100 g f.w.) registered a drop on day 3 for the MMC-AcA-SBEO- and HMC-AsA-GSEO-coated fruit compared to the control, followed by an increase on day 5 and another drop on day 7 matching the control (Treatment × Time, *p* < 0.0001*). On the other hand, when measured at 8 ℃ (20.75–23.76 mg/100 g f.w.), the HMC-AsA-GSEO maintained higher levels than the control toward the end of the study period (Treatment × Time, *p* < 0.0001*).

Despite the overall decrease in DM% in coated strawberries, along with the maintenance of a_w_ levels below that of the control, we noted that only the HMC-AsA-GSEO treatment exhibited a significant improvement in TPC together with AA (Figure 4a,b). In addition to the concentration of the solids, this effect might be ascribed to the more compact coating in comparison with the MMC treatments, which provided an enhanced protective barrier against polyphenol oxidative reactions [76,77]. Similarly, Dashipour et al. (2015) observed higher efficiency for TPC and AA in *Zataria multiflora* EO coatings [78]. 

Moreover, the incorporation of EOs has been considered to interact with the fruit tissue, as well as with the internal atmosphere; hence, it provides a suitable environment toward the inhibition of biochemical reactions and scavenging of hydroxyl radicals [79,80].

In this context, the oxidation/degradation of ascorbic acid is also affected, pertaining to a decelerated ripening process through the coating [74]. As presented above, our findings indicate that the vitamin C levels in the strawberry population differ as a function of coating treatment depending on time; we noted a significant effect of both of our main effects, coating and time, as well as their interaction with HMC-AsA-GSEO as the most efficient. It stands to reason that, despite the presence of EOs in all coating treatments, the HM chitosan is responsible for the delayed respiration rate and metabolic activity [76,79,81]. Similar results were observed by de Oliveira Filho et al. (2022) who analyzed strawberry samples during a 12 days period. The coated samples presented improved shelf life, antioxidant activity, phenolic compounds, and ascorbic acid content compared with uncoated strawberries. The coatings that contained essential oils proved to have antimicrobial activity [82]. In addition, an edible coating developed from chia seed mucilage and bacterial cellulose nanofiber was analyzed in order to determine its effect on the bioactive compounds and antioxidant enzyme activity of the strawberry samples. The coated strawberry samples proved to preserve the ascorbic acid content, protein content, and antioxidant activity, as well as the phenolic and flavonoid compounds [83]. In line with these, Martínez et al. (2018) described the protective effect of the *Thymus capitatus* essential oil incorporated in a chitosan edible coating on strawberries, delaying the reduction in antioxidant properties and extending the shelf life of the coated fruit to 15 days [45]. 

### 3.3. Effect of Edible Coating on Fruit Quality Parameters in Apples 

#### 3.3.1. Dry Matter and Water Activity

**Figure 5:**
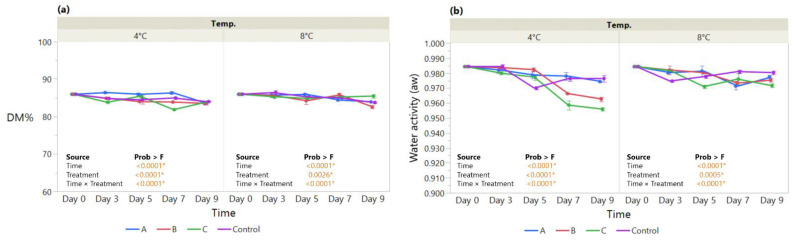
The effect of coating treatment on apple slices (**a**) Dry matter (DM%) and (**b**) Water activity (a_w_) during a 9-day storage period at 4 °C and 8 °C. The *p*-values for the effects of Treatment (coating), Time and Interaction Treatment × Time are shown. Data are means ± SEM (n = 3). Asterisk (*) indicates significance (*p* < 0.05*).

Observations were recorded for apple slices on the coating treatment they were assigned and their response for dry matter (%) and water activity (a_w_) for a period of 9 days (Figure 5). 

The assessment of the effect of the different coatings on DM% levels measured at both 4 °C and 8 °C indicates that there is a different effect of treatment depending on time (Treatment × Time, *p* < 0.0001* at 4 °C and 8 °C, respectively) (Figure 5a). For a 9-day storage assessment, the effect appears identical for the tested coatings, showing steady overall patterns (81.8–86.9%); for HMC-AsA-GSEO; however, a drop in DM% is delineated at day 7 (4 °C). 

For water activity, ranging between 0.96 and 0.98, the pattern of effect of time depending on the different coating treatments is more pronounced at 4 °C than at 8 °C (Figure 5b). The delayed decrease in a_w_ levels was noted only for MMC-AcA-SBEO and HMC-AsA-GSEO at 4 °C, whereas it manifested for all coating treatments at 8 °C when compared with the control. 

Unlike strawberries, apples contain fewer amounts of water. However, when sliced, it is only natural that moisture loss occurs [84]. The apparent improvement depicted through the tendency for a delayed weight loss (Figure 5a) is further substantiated when HMC-AsA-GSEO and MMC-AcA-SBEO (treatments C and B, respectively) exert a positive decrease in aw values at 4 °C (Figure 5b). It might be attributed to differences in the hydrophobicity of the coating treatments due to the addition of EOs. To a certain degree, a cross-linking effect might be responsible for a reduced interaction between the coating surface and the water molecules [74,76,78]. Like the case of strawberries, our findings on cold-stored apples indicate that coating permeability might be responsible for the plain overall improvement, revealed also at 8 °C. As a consequence of coating treatment application, a higher relative humidity surrounding the apple slices mitigates water loss [84]. 

#### 3.3.2. Total Polyphenol Content, Antioxidant Activity, and Vitamin C Content

**Figure 6:**
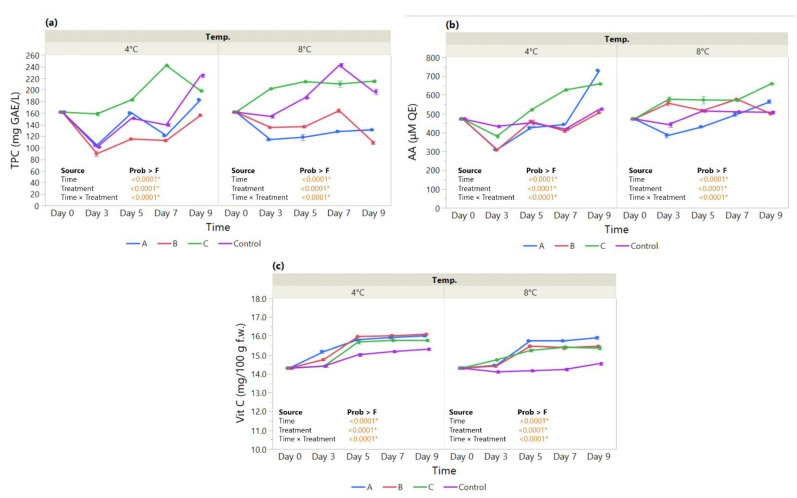
The effect of coating treatment on apple slices antioxidant status during a 9-day storage period at 4 °C and 8 °C. (**a**) Total Polyphenol Content (TPC—mg GAE/L); (**b**) Antioxidant Activity (AA—μM QE); and (**c**) Vitamin C content (Vit C—mg/100 g fresh weight). The *p*-values for the effects of Treatment (coating), Time and Interaction Treatment × Time are shown. Data are means ± SEM (n = 3). Asterisk (*) indicates significance (*p* < 0.05*).

The Least Squares Means Plot (Figure 6) shows the results of the test for the fixed factors, in this case, the two main effects for Treatment and Time. In addition, the interaction of Coating Treatment and Time is showing the differences by time broken apart by treatment. TPC levels measured at 4 °C (89–242 mgGAE/L) showed an increasing tendency for HMC-AsA-GSEO until day 7, after which it registered a significant drop below the control on day 9 (Figure 6a). By contrast, the two MMC coatings had contents lower than the control overall. As such, we can see a statistically significant effect for Treatment (*p* < 0.0001*) on TPC registered and for Time (*p* < 0.0001*) on TPC registered, as well as evidence that there is an interaction between Treatment and Time (*p* < 0.0001*). When evaluated at 8 °C (107–240 mg GAE/L), the evolution of TPC showed similar trends for the GSEO-treated fruit, with the HMC coating well above the MMC one, and higher than the control overall (Treatment × Time, *p* < 0.0001*). The MMC-AcA-SBEO did not manage to improve the TPC of apple slices over time, registering the lowest content at the end of the storage period. 

Our results show the AA levels drop at 4 °C for all treatments on day 3, followed by a progressive increase over time for the coatings containing GSEO (Treatment × Time, *p* < 0.0001*) (Figure 6b). The MMC-AcA-GSEO-coated fruit revealed a notable evolution in TPC, standing out at the end of cold storage, followed closely by HMC-AsA-GSEO (Treatment, *p* < 0.0001*). By increasing the storage temperature (8 °C), we observed different trends in the overall coating effect, as evidenced by the Treatment × Time interaction (*p* < 0.0001*). Nonetheless, by the end of the study period, a similar disposition in TPC was achieved, highlighted by the elevated levels of GSEO coatings compared with the control. 

Similarly, for the vitamin C content (Figure 6c), we evaluated whether there is anything about the effect of time that changes the effect of coating treatment. In this case, the effect of time is to have the vitamin C be constant, with a notable increase from day 3 observed at both 4 °C and 8 °C. Furthermore, the control fruit stored at 4 °C showed a slight increase; however, it was lower than the coated fruit. Nonetheless, when comparing the differences at each time point measured at 8 °C, the effect of treatment for the uncoated apple slices is the same at all time periods. 

Present in variable concentrations in apples, phenolic compounds together with ascorbic acid, account to a great extent for the antioxidant activity of food and promote human health [85,86]. Minimally processed apples are accompanied by an enzymatic synthesis of polyphenols through the activity of phenylalanine ammonia lyase (PAL); at the same time, polyphenol oxidase (PPO) causes the degradation of phenolic compounds [85,87]. In addition, the content of acidic compounds during ripening has a decreasing tendency over time, which might be attributed to the oxidation of organic acids along with the rise in pH values [84,88]. The tandem use of edible coatings and antioxidants (essential oils) is able to inhibit these degradation processes [87,89]. In this context, our findings suggest that the incorporation of GSEO into the HMC coating enabled the stabilization of pH and decreased the activity of PPO, which in turn contributed to the higher TPC and AA monitored at both 4 °C and 8 °C. Given that gas permeability reduction is promoted by using edible coatings, fruit are accompanied by a decrease in respiration rate during cold storage; accordingly, ascorbic acid degradation is also diminished [86]. Accordingly, the overlapping patterns for vitamin C (Figure 6c) provide further insight on the considerable improvement pertaining to the antioxidant activity levels. It can be inferred that the assessed coatings are not interacting with the vitamin C content, irrespective of the treatment, and are able to maintain this parameter above the physiological postharvest levels. Similar results were obtained when rose apples (*Cv. Tabtimchan*) were coated with a sodium alginate-based edible coating in order to maintain the postharvest quality when stored at low temperatures. The coated samples had a significantly reduced respiration rate and weight loss throughout the 10 days of storage. The total phenolic content and antioxidant activity presented higher values compared to the control on day 10 [90]. In addition, Kumar et al. (2018) reported the improvement of shelf life of fresh-cut Royal Delicious apple wedges when applied an edible coating containing antibrowning agents. Their application indicated a significant effect on the apples’ weight loss and enzymatic browning and retarded microorganism growth during a 7-day storage period at 5 ± 2 °C [91]. In this context, similar findings were indicated by Farina et al. (2020) when lemon essential oil was incorporated in aloe vera gel-based edible coatings. The postharvest quality of fresh-cut Fuji apples was improved in terms of soluble solids, titratable acidity, and pH and managed to maintain the overall nutrients and vitamin content throughout storage [92].

### 3.4. Microbiological Analysis

**Figure 7:**
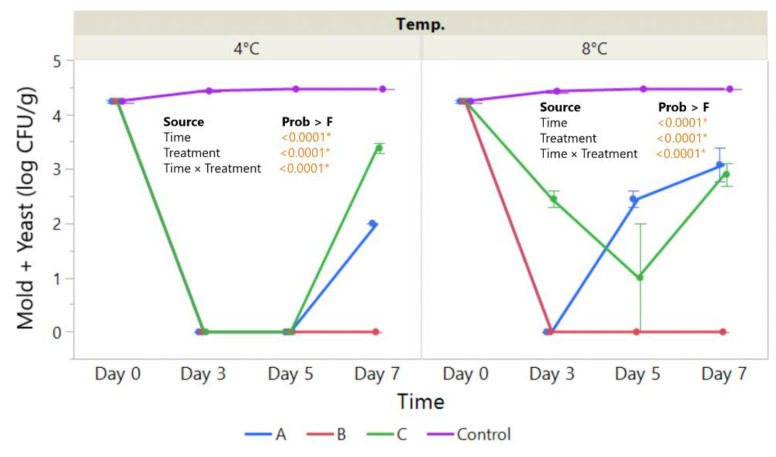
The effect of coating treatment on total number of strawberry mold and yeast colonies (log CFU/g) for a 10^−2^ dilution during a 7-day storage period at 4 °C and 8 °C. The *p*-values for the effects of Treatment (coating), Time and Interaction Treatment × Time are shown. Data are means ± SEM (n = 2). Asterisk (*) indicates significance (*p* < 0.05*).

Strawberries are fruits that have a short shelf life because of their high water content and high physiological activities, thus being susceptible to several types of microorganisms, the main one being *Botrytis cinerea* [56]. The results of the microbiological analysis of yeasts and molds are quite straightforward; the microbial load was significantly decreased throughout the storage period at both temperatures, 4 °C and 8 °C, as shown in Figure 7. All types of coatings, compared to the control samples that were uncoated, had significantly lower levels of microbial load. However, strawberries coated with chitosan, acetic acid solutions, and sea buckthorn oil provided the lowest count of molds and yeasts until day 7 of the analysis. The results are in accordance with the lower water activity presented in this study by the coated samples, thus providing an unsuitable environment for microbial growth. These results are in agreement with those obtained by Xin et al. (2022) [93], Lukša et al. (2018) [94], and de Mata et al. (2022) [95] who studied the antifungal effect of sea buckthorn and grape seed EOs, and their findings confirmed their antifungal activity over several fungi strains. Moreover, Velickova et al. (2013) [96] show that chitosan-based edible films present antimicrobial growth and extend the shelf life of strawberries.

In the case of the microbiological assay of the apple samples, it is noted that no microbial load was observed at the tested dilutions. 

### 3.5. Study Limitations

The present study was intended to stand as a potential tool in support to maintaining the postharvest quality of fruit. Yet, given the nature of the employed methods, some drawbacks of our study include (a) missing sensorial evaluation and (b), arguably, a lack of measurements of the tested parameters at more realistic temperatures (e.g., 23 °C). Nonetheless, taking into account the adjunction of functional ingredients pertaining to added-value features (e.g., antimicrobial activity), edible coatings represent a suitable choice towards maintaining the characteristics of fresh-cut fruit, a well-established challenge for food processors. 

In order to attain adequacy from the economical perspective and to provide pertinent insight toward real-life applications, consistent research and development on edible coating methods is needed [7]. Moreover, consumers are progressively demanding healthy foods and become more educated; consequently, from a practical perspective, one of the approaches employed to extend the storability of perishable produce is the application of edible coatings, followed by cold storage, deemed more realistic than at room temperature [30,31,32].

With regard to the taste of the coated fruit, we did not evaluate the sensorial attributed by employing a trained panel. The addition of ascorbic or acetic acid along with the sea buckthorn or grape seed essential oils may indeed have the potential of altering the taste of the resulting coated fruit. However, given that postharvest losses of fruits are due to fungal infection, physiological disorders, and physical injuries, edible coatings can be used as a protective barrier to reduce respiration rates, retard microbial growth, and improve texture quality [30,31,32]. As such, if the coating film applied herein were to be unable to maintain the firmness and microbial load of the samples after refrigerated storage, off-odor/off-flavor may have indeed occurred. In addition, the use of essential oils as antimicrobial agents prolonged the desirable appearance of the tested fruit and retarded the development of microorganisms; these adjuvants have been previously deemed acceptable for consumers [3,7]. In a similar manner, Pavinatto et al. (2020) observed that a chitosan/glycerol 30% coating had an insignificant effect on the flavor, appearance, aroma, and texture of coated strawberries [55].

A fair assumption would be that the measurements could seem short and some results scattered and perceived as systematic errors, which may not be representative enough. Nonetheless, having achieved mechanical/biological protection/extension afforded herein by the coating in conjunction with the inclusion of essential oils, with no disproportionate response of the measurements corroborated with other scientific works, the benefits of the proposed approach would be desirable. Moreover, having assumed a proper tandem assessment of the physicochemical parameters at two different temperatures of cold storage (4 °C and 8 °C), our observations could provide supplementary information as to what extent the functional coatings can explain the GSEO and SBEO potential and render the measurements of the study relevant pertaining to the retention of postharvest quality. Taking into account both the drawbacks and the strengths of the study, these observations warrant their practical relevance for the purpose at hand and merit further exploration with regard to their potential application. 

## 4. Conclusions

The developed chitosan-based and EO edible coatings managed to improve the postharvest quality and shelf life of the strawberries and apple slices during the storage period, minimizing physicochemical changes and maintaining a significantly lower microbial growth compared to the uncoated control samples. Coating strawberries and apples slices with the three chitosan-based edible coatings presented a beneficial impact in preserving the ascorbic acid, total phenolic content, and antioxidant activity in fruits during the cold storage period. Strawberries and apples coated with HMC-AsA-GSEO presented the best results regarding the ascorbic acid content and the antioxidant activity during the shelf-life period. In the case of strawberry samples, after 7 days of cold storage, the chitosan-based edible coatings functionalized with essential oils were able to reduce the microbial growth significantly, thus maintaining their postharvest and shelf life. It can be concluded that the developed chitosan-based coatings represent a promising technique to attain mechanical protection and preserve the postharvest quality parameters and shelf life of strawberries and apple slices during cold storage.

## Figures and Tables

**Table 1:** Composition of the obtained chitosan-based edible coatings.

Coating	Code	Composition
A	MMC-AcA-GSEO	2% Medium molecular weight chitosan, 1% acetic acid, grape seed EO
B	MMC-AcA-SBEO	2% Medium molecular weight chitosan, 1% acetic acid, sea buckthorn EO
C	HMC-AsA-GSEO	1% High molecular weight chitosan, 2% ascorbic acid, grape seed EO

## Data Availability

The authors confirm that the data supporting the findings of this study are available within the article.
